# ALKBH family members as novel biomarkers and prognostic factors in human breast cancer

**DOI:** 10.18632/aging.204231

**Published:** 2022-08-17

**Authors:** Hongxi Chen, Lei Zhou, Juanni Li, Kuan Hu

**Affiliations:** 1 Department of Gastrointestinal Surgery, Hunan Provincial People’s Hospital (The First Affiliated Hospital of Hunan Normal University), Changsha 410005, Hunan, China; 2 Department of Anesthesiology, Third Xiangya Hospital of Central South University, Changsha 410008, Hunan, China; 3 Department of Pathology, Xiangya Hospital, Central South University, Changsha 410008, Hunan, China; 4 National Clinical Research Center for Geriatric Disorders, Xiangya Hospital, Central South University, Changsha 410008, Hunan, China; 5 Department of Hepatobiliary Surgery, Xiangya Hospital, Central South University, Changsha 410008, Hunan, China

**Keywords:** ALKBH, breast cancer, expression, prognosis, immune infiltration

## Abstract

Breast cancer is the most common lethal carcinoma worldwide and better targeted therapies are still worthy of exploration, having had some great successes already. Abnormal expression of ALKBH members were found in various cancers, and the roles played by it were the focus of attention. The ALKBH gene family encodes nine homologous enzymes (ALKBH1-8 and FTO) to repair DNA or RNA depending on Fe2+ and α-ketoglutarate (α-KG), which is related to carcinogenesis. In this study, we applied several databases to explore the roles of ALKBHs in breast cancer. We found that ALKBH members were abnormal expression in breast cancer and associated with tumor stage and subclasses. Higher alteration rates of ALKBH family were found in breast cancer. Function enrichment revealed that several cancer-associated signal pathways were related to ALKBH family such as PI3K-Akt signaling pathway and axon guidance. Infiltration of immune cells (Eosinophiles, NK CD56bright cells, mast cells, T helper cells and so on) were strongly related to ALKBHs. Moreover, we further found that there was strong correlation between ALKBH7 and higher age, later T stage, ER/PR positive and post-menopause of breast cancer patients, and patients with higher ALKBH7 expression had shorter overall survival (OS) and post progression survival (PPS). In conclusion, our findings may provide novel insights into ALKBH-targeted therapy for breast cancer patients, and ALKBH7 may be a potential prognostic biomarker.

## INTRODUCTION

Breast cancer is one of the most lethal carcinomas worldwide, even though progress has stagnated reported by the American Cancer Society recently [1, 2]. There is a significant difference in the survival rate worldwide. It has been estimated that 80% of patients could survive beyond 5 years in developing countries, which was better than 40% in developing countries. According to the character of biomarkers, breast cancer is divided into three subtypes: hormone receptor-positive/ERBB2 negative, ERBB2 positive and triple negative. Different subtypes may refer to prognosis. Triple negative may be better than others [3, 4]. Surgery, chemotherapy, and targeted therapy are all effective treatments for the breast cancer patients. Targeted therapy is a successful treatment, especially for growth factor receptor 2 positive (HER2+) breast cancer, other targets were also found consciously [5–8].

The ALKBH family as a kind of demethylase is detected in various cancer and is associated with carcinogenesis, and may be a potential target for novel anticancer therapy [9, 10]. The ALKBH family consists of nine homologous enzymes (ALKBH1-8, FTO) whose catalytic activity depends on Fe2+ and α-ketoglutarate (α-KG). Even though the paralogs share a homologous catalytic core, different combinations of substrates lead to different functions, which are obviously characteristic of the ALKBH family [11].

DNA alkylation damage often occurs in environmental chemicals and chemotherapy, the repair of which is particularly important for DNA repair and prevents the cytotoxic or mutagenic effects of various types of DNA lesions with further carcinogenesis [12]. ALKBH family may play a pivotal role in DNA repair of alkylation damage as the previous study, but it is almost blank about the role of the ALKBH family in breast cancer [13]. This research is aimed to explore the role played by the ALKBH family in breast cancer.

## RESULTS

### Differential expression of ALKBH family in breast cancer

**Figure 1:**
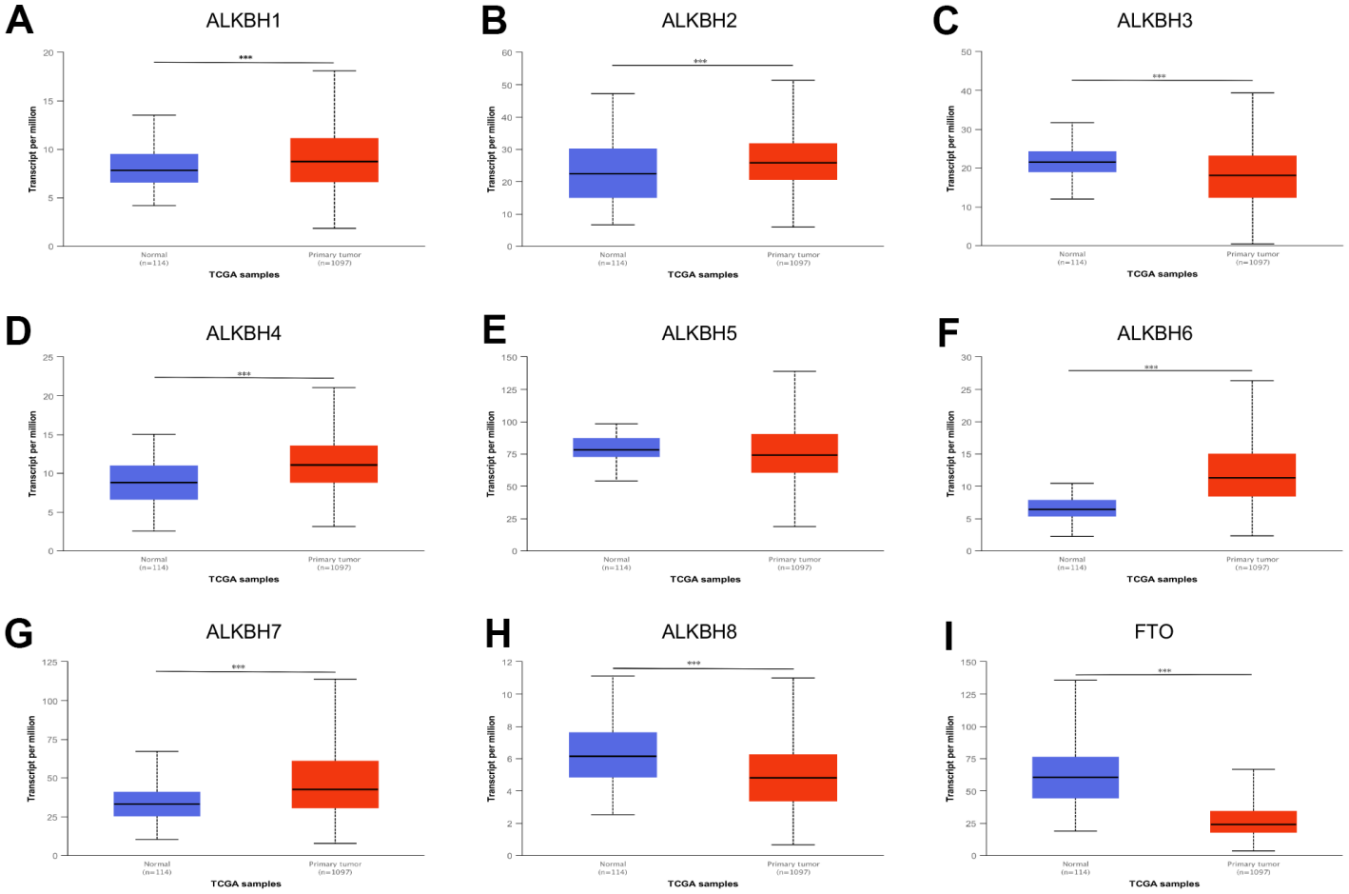
**The expression profile of ALKBH family in BRCA.** (**A**–**I**) ALKBH1-8 and FTO mRNA expression levels between normal tissues and BRCA tissues (UALCAN). *: P<0.05, **: P<0.01, ***: P<0.001.

We performed breast cancer analyses to compare the mRNA expression of nine homologous enzymes between the primary tumor samples and normal samples by using UALCAN. It was to be found only ALKBH5 have no significant differential expression, and other homologous including ALKBH1, ALKBH2, ALKBH3, ALKBH4, ALKBH6, ALKBH7, ALKBH8, FTO all had differential expression mRNA. With compared the expression of ALKBH family members in breast cancer and normal samples, the up-regulation was ALKBH1, ALKBH2, ALKBH4, ALKBH6, ALKBH7, and others such as ALKBH3, ALKBH8, FTO was down-regulation (Figure 1).

### ALKBH family are associated with tumor stage and subclasses

**Figure 2:**
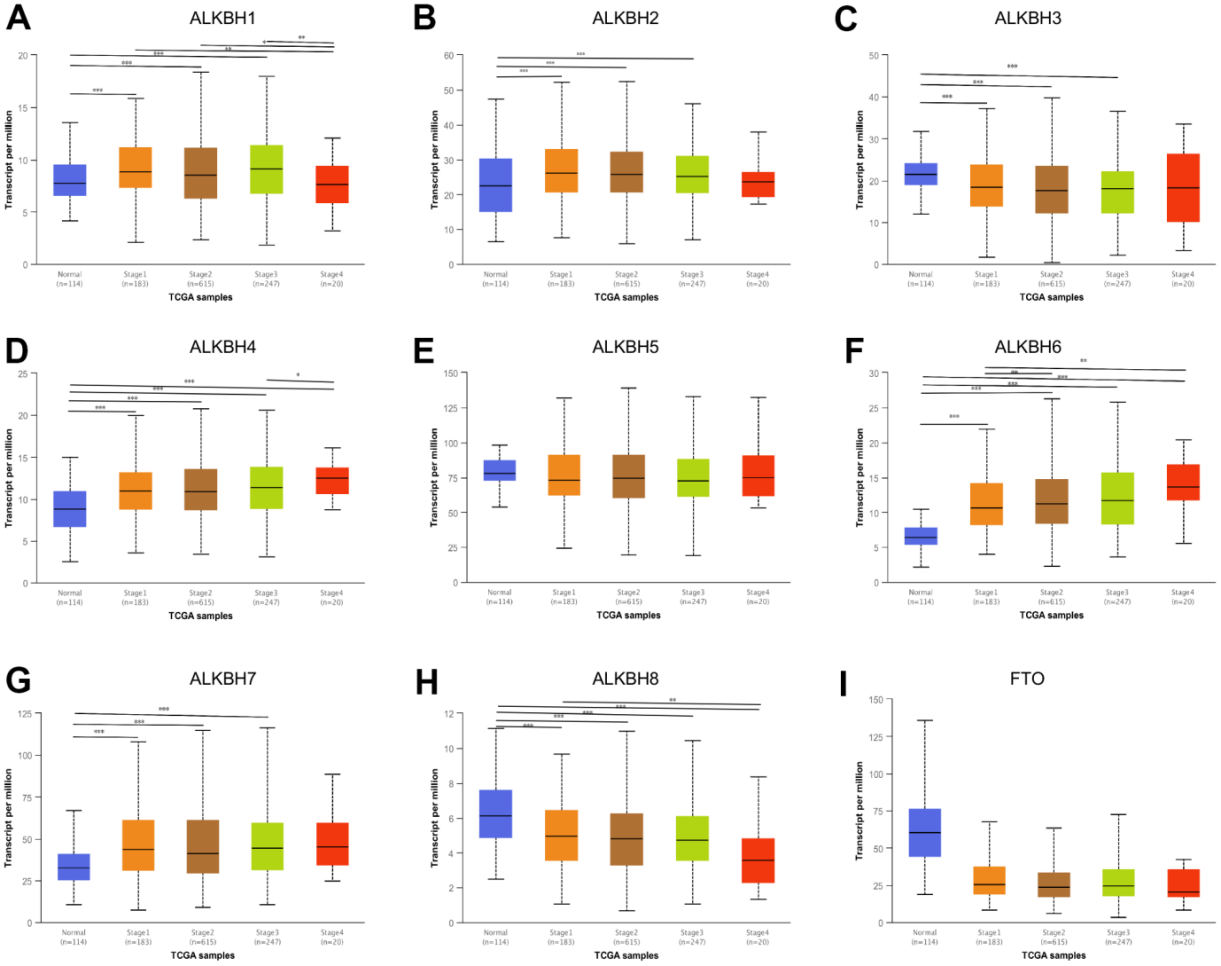
**The correlation between the expression of ALKBH members and individual cancer stages.** (**A**–**I**) The relationship between ALKBH1-8 and FTO mRNA expression levels and individual cancer stages (UALCAN). *: P<0.05, **: P<0.01, ***: P<0.001.

Compared to the normal tissue, we found almost all ALKBH homologous were associated with tumor stage except ALKBH5 and FTO. On account of the advance stage which indicates progression, ALKBH1, ALKBH4, ALKBH6, and ALKBH8 may be the potential biomarker for predicting tumor progression. The result further showed up-regulated homologous ALKBH1, ALKBH4, and ALKBH6 in the advance stage, while the down-regulated gene was ALKBH8 (Figure 2).

Breast carcinoma is classified into various subclasses such as luminal type, HER2 positive, and triple negative breast cancer (TNBC), different subclasses may have different clinical implications [14–16]. In all of the ALKBH family member associated with breast cancer subclasses, different ALKBH substrates show differential expression in three subclasses in our research (Supplementary Figure 1).

### Genetic alteration of the ALKBH family in breast cancer

**Figure 3:**
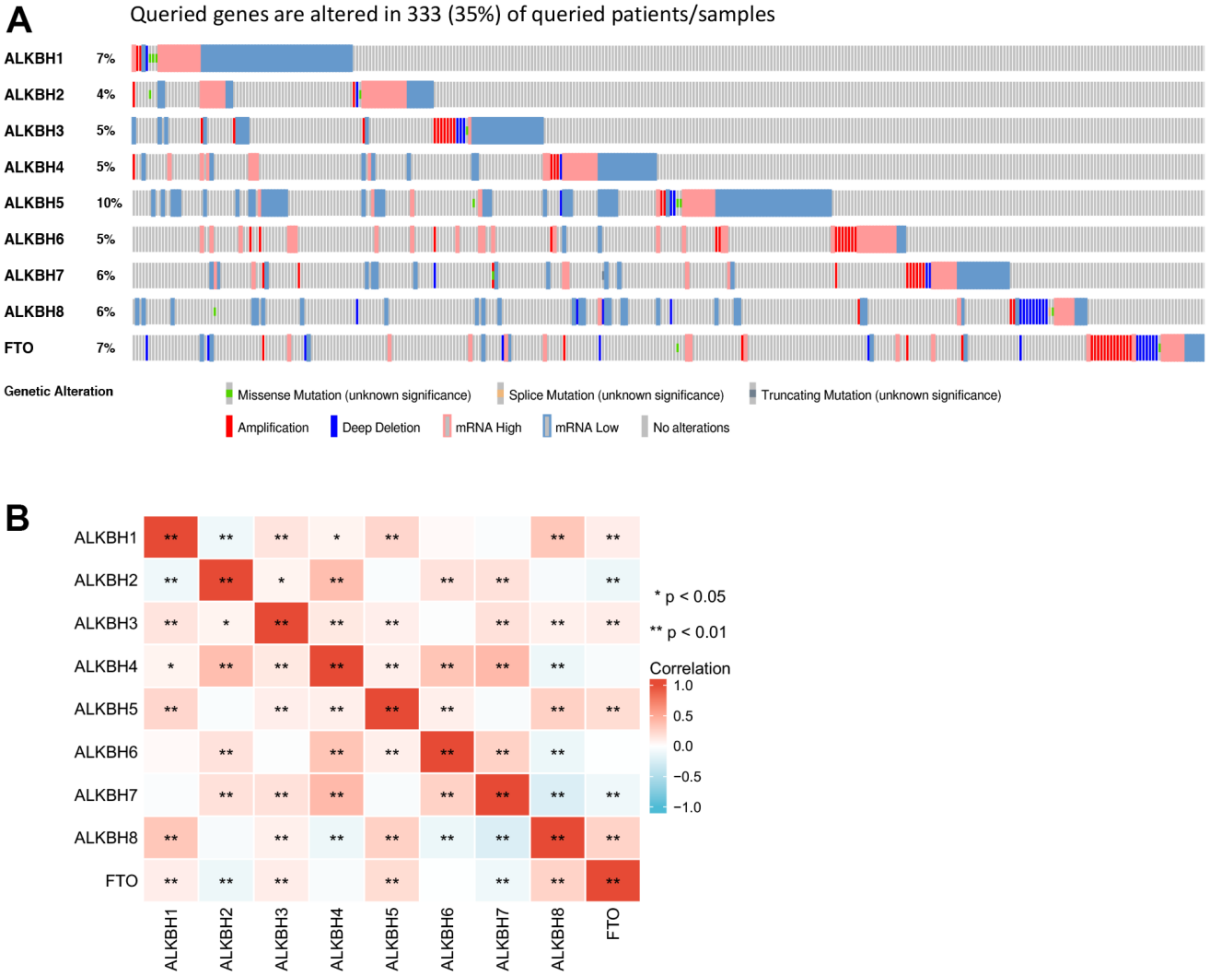
**The gene alteration rate and the correlation with different ALKBH members.** (**A**) the frequency and types of genetic alterations of different ALKBH family members in BRCA performed by cBioPortal (333 patients). (**B**) the correlations between each ALKBH member (GEPIA). *: P<0.05, **: P<0.01, ***: P<0.001.

For further analyzing the profile of the ALKBH family genetic alteration, we investigated the alteration by utilizing the cBioPortal database. The most frequent of alteration was ALKBH5 (10%). While the lowest was ALKBH4 which was about 4%. Queried ALKBH genes are altered in 333 queried patients which means the frequency of total alteration is 35% (Figure 3A). In addition, the most significant correlation substrate was shown in Figure 3B, which may predict the different combination of substrates for the ALKBH family (Figure 3B and Supplementary Table 1).

### Functional enrichment of ALKBH family

**Figure 4:**
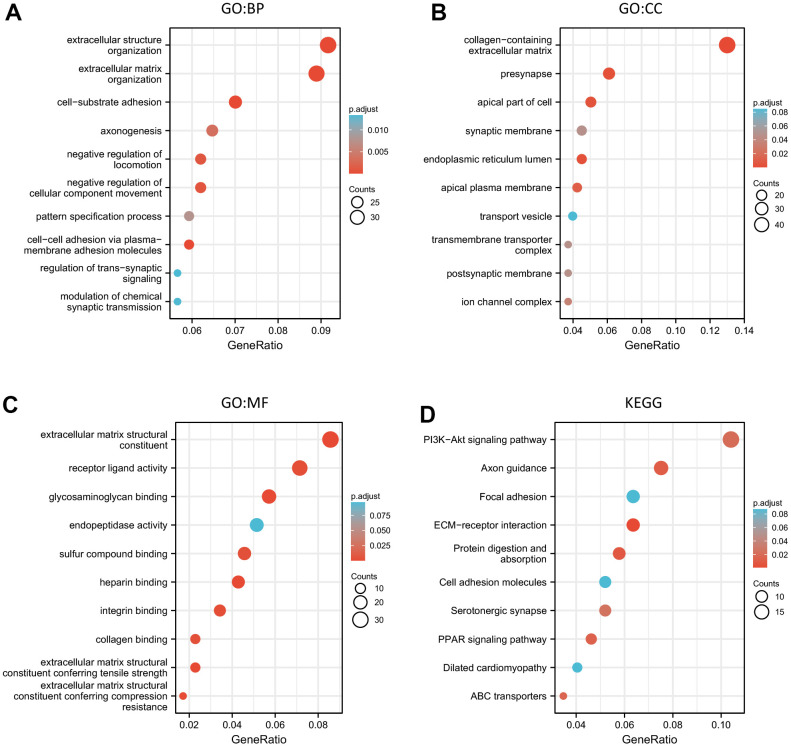
**The functional enrichment analysis of the ALKBH family associated co-expressed molecules in breast cancer.** (**A**–**C**) GO (BP, CC, and MF) analysis of the ALKBH family associated molecules. (**D**) KEGG pathway analysis of the ALKBH family associated molecules.

In our study, the result of functional enrichment of the ALKBH family was as follows: Firstly, extracellular structure organization was the most highly enriched biological process (BP). Secondly, the collagen-containing extracellular matrix was also highly enriched in the cellular component (CC) categories. Thirdly, in the molecular function (MF) categories, extracellular matrix structural constituent was most highly enriched and followed by receptor-ligand activity, and glycosaminoglycan binding. Finally, the KEGG pathway results showed that the potential function of the ALKBH family may be linked to the PI3K-Akt signaling pathway, Axon guidance, ECM-receptor interaction, protein digestion, and absorption, PPAR signaling pathway (Figure 4 and Supplementary Tables 2, 3).

### Immune cell infiltration of the ALKBH family

**Figure 5:**
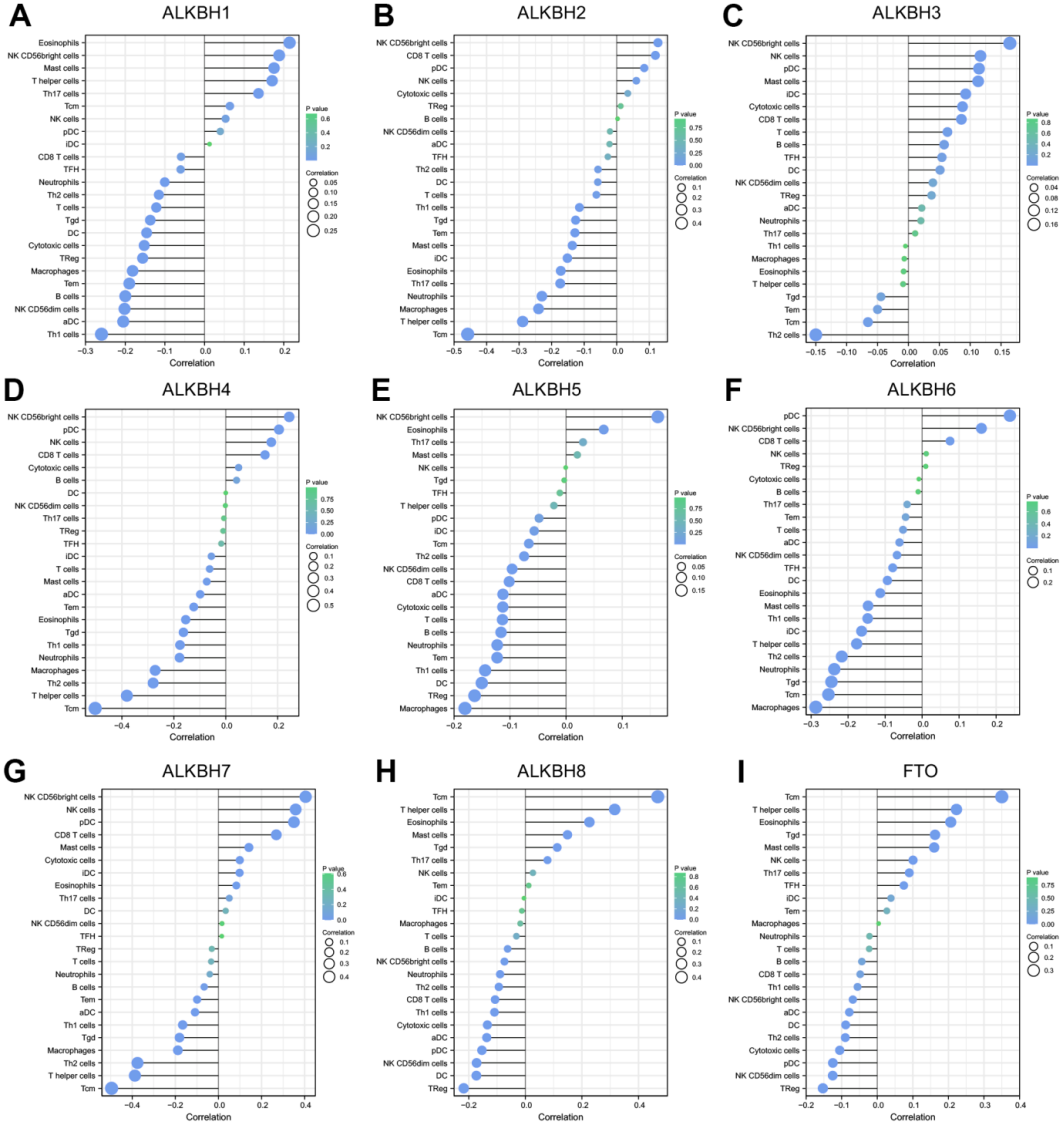
The correlation between the infiltration levels of various immune cells and the expression of ALKBH members including (**A**–**I**) ALKBH1-8 and FTO.

For studying the relationship between immune cell infiltration and the expression profiles of the ALKBH family in breast cancer, we extracted the data from the TCGA-BRCA (breast invasive carcinoma) project. The expression of ALKBH1 was positively linked to Eosinophils, NK CD56 bright cells, mast cells, and T helper cells, whereas it had a negative relationship with the infiltration of Th1 cells, aDC (activated DC). ALKBH2 was most negatively linked to Tcm (T central memory). ALKBH3 was further positively relationship with NK CD56 bright cells, and NK cells and was negative with Th2 cells. ALKHB4 was a negatively relationship to Tcm which was similar to ALKBH2. ALKBH5 was positively linked to NK CD56 bright cells and negatively to Macrophages, TReg, DC. ALKBH6 was positive correlation to pDC (Plasmacytoid DC) and was negative to Macrophages and Tcm. ALKBH7 was positively related with NK CD56 bright cells and negatively related with Tcm. ALKBH8 and FTO were both positively linked to Tcm. In brief, there was a closely relationship between ALKBH family and immune cells infiltration (Figure 5).

### The prognostic value of the ALKBH family in patients with breast cancer

**Figure 6:**
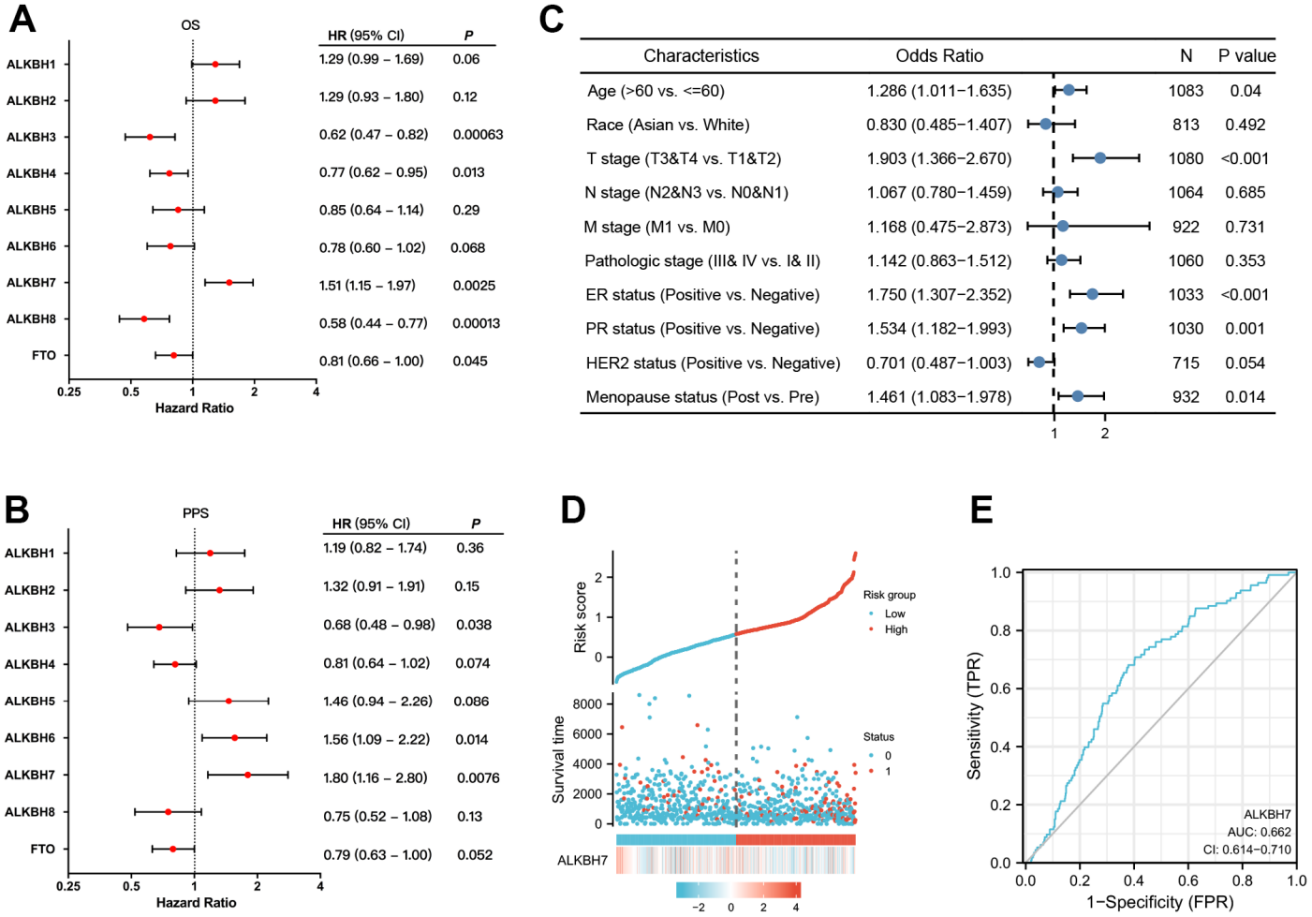
**The prognostic value of ALKBH members in BRCA.** (**A**, **B**) The correlations between ALKBH members and OS and PPS in patients with BRCA. (**C**) The relationship between clinicopathologic features and ALKBH7. (**D**) The high expression of ALKBH7 may lead to a higher death rate. (**E**) The sensitive biomarker to evaluation (AUC:0.662).

We evaluated the prognostic value of the ALKBH family for patients with breast cancer by Kaplan-Meier plotter. As the data shown, the OS of breast cancer patient was significant difference when gene expression was changed in ALKBH3 (p=0.00063), ALKBH4 (p=0.013), ALKBH7 (p=0.0025), ALKBH8 (p=0.00013) and FTO (p=0.045) (Figure 6A). While the PPS was significant difference in patient with changed gene expression in ALKBH3 (p=0.038), ALKBH6 (p=0.014) and ALKBH7 (p=0.0076) (Figure 6B). Based on the above results, we found that only the ALKBH7 expression trend was consistent with its prognostic result of OS and PPS.

Next, we analyzed the correlation between clinical characteristics and ALKBH7 expression in breast cancer patients and found that higher expression of ALKBH7 was related to higher age, later T stage, ER-positive, PR-positive, and post-menopause (Figure 6C). As the risk factor diagram showed, when the risk score increased, the number of dead was increasing accordingly. The higher expression of ALKBH7 was relative to a shorter survival time (Figure 6D), and the sensitivity (TPR) is also higher with AUC approached 1 (AUC=0.662) (Figure 6E).

## DISCUSSION

It is about 40 years since the ALKBH family was founded. Firstly, it has been discovered in *E. coli* cells.

Gene AlkB encodes Escherchia coli AlkB protein to protect against alkylating agents acting as a one-protein repair system. ALKBH family is the AlkB homologs, which act on a diverse spectrum of specific substrates and involve many functions such as DNA repair and regulatory role. But there is still to study the evolutionarily conserved function of ALKBH protein and their regulatory impact on various cellular processes [17]. Even now there is still a lack of evidence to support that ALKBH paralogs stand out as a high-frequency pan-cancer gene. ALKBH proteins could reverse N-methylation in DNA and RNA, and play potential roles in cancers as a group [18, 19]. Recently an increasing body of evidence suggested that the ALKBH family promoted tumorigenesis and might be a potential target for novel anticancer therapy [20–22]. There are nine members of the ALKBH family in humans. It is necessary to point out that their roles in breast cancer are still poorly understood. Through our research, almost all of them were differentially expressed in breast cancer samples except ALKBH5. In contrast, Chuanzhao Zhang et al. group had confirmed that Hif-dependent ALKBH5 expression mediates enrichment of breast cancer stem cells (BCSC) in the hypoxia tumor microenvironment by reducing numbers of BCSCs by knockdown of ALKBH5 expression [23]. Given later test not being derived from the human organization, further test needs to verify. It is short of direct proof about the relationship between ALKBH family and stage or subclasses in breast cancer, this study provided that ALKBH1, ALKBH4, and ALKBH6 might be associated with later stage, while ALKBH8 was relative to an earlier stage. The expression of ALKBH homologous was correlated with subclasses, which reminded us of the tumor heterogeneity.

The total genetic alteration of the ALKBH family was 35%, and the ALKBH5 mutation rate was the highest, which was only 10%. It was similar to support that any ALKBH paralogs could not stand out as a high-frequency pan-cancer. In addition, the degree of correlation between different ALKBH paralogs was different and maybe because of different roles played by different substrate combinations [24–26]. As a group, what was the ALKBH family’s role in breast cancer? It was a pity that there was limited data on ALKBH family function in breast cancer, but we found that ALKBH family abnormal expression was associated with PI3K-AKt signaling pathway, Axon guidance, ECM-receptor interaction, protein digestion, and absorption, PPAR signaling pathway by performing functional annotation base on GO and GESA. PI3K-AKT is a signaling pathway involved in cell proliferation, survival, invasion, and migration which is a key character of tumor growth, especially since up to 40% of PIK3CA mutations are estrogen receptor(ER) positive and human epidermal growth factor receptor2 (HER2)-negative in primary and metastatic breast cancer. Meanwhile, 20-30% of breast cancers appear HER2 over-expressing, which may lead to PI3K-AKT activation [27]. In terms of the regulatory mechanism, proliferating cell nuclear antigen (PCNA) known as a nuclear protein essential for the regulation of DNA repair may play a pivotal role, because the ALKB homologue 2 PCNA interacting motif (APIM) was found to be associated with the PI3K-AKT pathway [28]. ECM-receptor interaction was considered to be closely related to breast cancer, especially inferior breast cancer survival [29, 30]. Ferroptosis is an iron-dependent cell death mode with the intracellular accumulation of reactive oxygen species (ROS), and dysregulation of iron metabolism can lead to tumorigenesis. A recent study demonstrates that Fe2+-based metal-organic skeleton with transferring Fe2+ to cancer cells triggers a Fenton reaction, which produces excess ROS, and induces ferroptosis in the breast. PPARα activity is necessary for the promotion of ferroptosis in cancer by MDM2 and MDMX (p53 negative regulators) [31]. In some situations, PPARαwill regulate ROS signal way to lead disease, PPARα agonist, MHY3200, alleviates renal inflammation during aging via regulating ROS/Akt/FoxO1 signaling [32]. ROS accumulation and IGF-IR inhibition contribute to fenofibrate/PPARα-mediated inhibition of glioma cell motility *in vitro* [33]. Manganese superoxide dismutase deficiency triggers mitochondrial uncoupling and the Warburg effect [34].

Increasing results of preclinical trials and recent clinical data verify an immunotherapeutic strategy may be the important treatment means in breast cancer, and immune infiltration in the tumor immune microenvironment (TIME) predicts the prognosis of BC as accumulating evidence supports. The number of T cells may have a significant association with the prognosis in patients with triple-negative breast cancers. So, immune cell infiltration (ICI) patterns play a vital role in tumor progression in breast cancer [35]. Immune cell infiltration and ECM stiffness may promote breast cancer invasion and aggression. I. Acerbi et al. team have found that the greatest number of infiltrating macrophages and the highest level of TGF-β signaling within the cells at the invasive front, in addition, stroma stiffness and the level of cellular TGF-βsignaling were positively related to each other, and infiltrating tumor-activated macrophages, which was correlated with tumor subtypes [36]. Deserve to be mentioned, NK CD56 bright cells were positively related to ALKBH7, which proved to be correlated with CXCL12 expression in a pancreatic pre-tumor cell model and MARCKS expression in HCC, meanwhile, all of them expressed in various tumor [37, 38]. CXCL12 expression and activated hepatic stellate cells (aHSCs) abundance were associated with breast cancer patients with liver metastases, the interplay between NK cells and aHSCs master switched breast cancer dormancy to outgrowth [39]. MARCKS from plasma extracellular vesicles is also considered to diagnose breast cancer [40]. But there was a lack of directing proof to definite whether the connection between ALKBH7, NK CD56 bright cells, and CXCL12 or MARCKS or not. Central memory T cell (Tcm) play a critical important role in tumor immunity and was always a connection with favorable prognosis in various tumors, meanwhile, Deng, L et al. pointed out that Tcm enrichment might be related to negative prognosis in breast cancer, higher expression of ALKBH7 might predict worse prognosis as our research [41].

ALKBH7 was likely to be related to a negative prognosis in breast cancer as our aforementioned analysis by immune cell infiltration result. For exploring the prognostic value of the ALKBH family, we analyzed survival time data from TCGA by Kaplan-Meier plotter and found the correlation between OS or PPS with ALKBH expression. High expression of ALKBH7 was associated with poorer OS and PPS, which meant a worse prognosis. In addition, higher expression of ALKBH7 might be associated with higher age, later T stage, ER-positive, and post-menopause, which also supported the conclusion of worse prognosis, and the high sensitivity made the ALKBH7 be optimum predict prognosis in the breast. Except for our research, there was a lack of direct proof to support ALKBH7 as a biomarker with a poor prognosis up to now. As one of α-ketoglutarate-dependent dioxygenases, ALKBH7 expression was positively associated with D-2-hydroxyglutarate dehydrogenase(D2HGDH) as Gene expression profile data and later is considered a potential prognostic marker in breast cancer [42]. Disorder of ALKBH family level in mammals induce many types of diseases including cancer and may serve as potential cancer markers as increasing proof [43].

This study explored the roles of nine ALKBH family members in breast cancer in several aspects including genetic alteration, expression, function, immunity, tumor stage and subclasses, which gave us an integrated insight into the ALKBH family members in breast cancer. However, several limitations still need to be pointed. Firstly, our research was conducted basically using several online databases and analytic tools, solid experiments were needed to obtain more credible results, not just these conclusions from these bioinformatic analyses. This is our next step to conduct the cell biology experiments and clinical verification, especially to investigate the role of ALKBH7 in breast cancer. Moreover, we need explore advanced mechanisms for a better understanding of the value of ALKBH family members in diagnostic and therapeutic role in breast cancer.

## CONCLUSIONS

As our group data have shown, abnormal expression of the ALKBH family was associated with breast carcinogenesis, and tumor stage and subclasses were both linked to ALKBH expression. ALKBH genetic alteration might cause immune cell infiltration by activating correlated signaling pathways such as the PI3K-AKT pathway, PPAR pathway, and so on, which induce breast carcinogenesis in finally. In addition, higher ALKBH7 expression was likely to relate to the poor prognosis for patients with breast cancer and might be a potential breast cancer biomarker and possible therapeutic target in the future.

## MATERIALS AND METHODS

### UALCAN

ALKBH expression profiles and their association with clinical parameters were collected from UALCAN (http://ualcan.path.uab.edu/index.html, including 114 normal tissue and 1097 primary tumor) [44]. Boxplots were generated to calculate differential expression of ALKBH, as compared to normal and tumor.

### GEPIA

Gene Expression Profiling Interactive Analysis (GEPIA) is a database which is designed to help users to understand gene expression on the whole (http://gepia.cancer-pku.cn/) [45]. We evaluate the correlations among the individual ALKBH family members in breast cancer tissue by using “Correlation Analysis” model in GEPIA. P-value is settled at 0.05.

### Kaplan-Meier plotter

The Kaplan-Meier plotter is used to assess the relationship between gene expression and the survival trend of various cancer patients (http://kmplot.com/analysis/) [46]. We evaluated the prognosis of breast cancer patients by means of overall survival (OS) and post progression survival (PPS) curves in our study. In addition, we verified the high expression and low expression groups from the figures, and it has statistical significance if the p-value<0.05.

### cBioPortal

cBioPortal includes data about 200 cancer genomics and provides a user-friendly analysis strategy concerning gene-disease associations (http://www.cbioportal.org/) [47]. In our study, we analyzed the genetic alterations of the ALKBH family in breast cancer tissues by searching cBioPortal.

### XianTao

XianTao aims to provide users with a better understanding of gene interpretation, and enrichment results to the researcher (https://www.xiantao.love) [48–50]. In this study, we get several enrichment pathways associated with the ALKBH family in breast cancer disease from the database. Gene Ontology (GO) and the Kyoto Encyclopedia of Genes and Genomes (KEGG) are the main two enrichment pathways. Meanwhile, the relationship between ALKBHs expression and the infiltration of immune cells was also applied by using the TCGA-BRCA project through XianTao tool.

### Statistical analysis

The transcriptional expression level between tumor and normal tissues was analyzed using Student’s t-test. The survival value was performed by Kaplan-Meier analysis. The relationship between clinicopathologic features and ALKBH7 was analyzed by Logistic regression. p < 0.05 was statistically significant.

## Supplementary Material

Supplementary Figure 1

Supplementary Table 1

Supplementary Table 2

Supplementary Table 3
