# Supplementary material for: ALKBH family members as novel biomarkers and prognostic factors in human breast cancer
Source: Aging (Albany NY). 2022 Aug 17;14(16):6579–93. doi: 10.18632/aging.204231 (PMC9467415; doi:10.18632/aging.204231)
Supplement: Supplementary Figure 1 [file aging-14-204231-s001.pdf]

SUPPLEMENTARY FIGURE

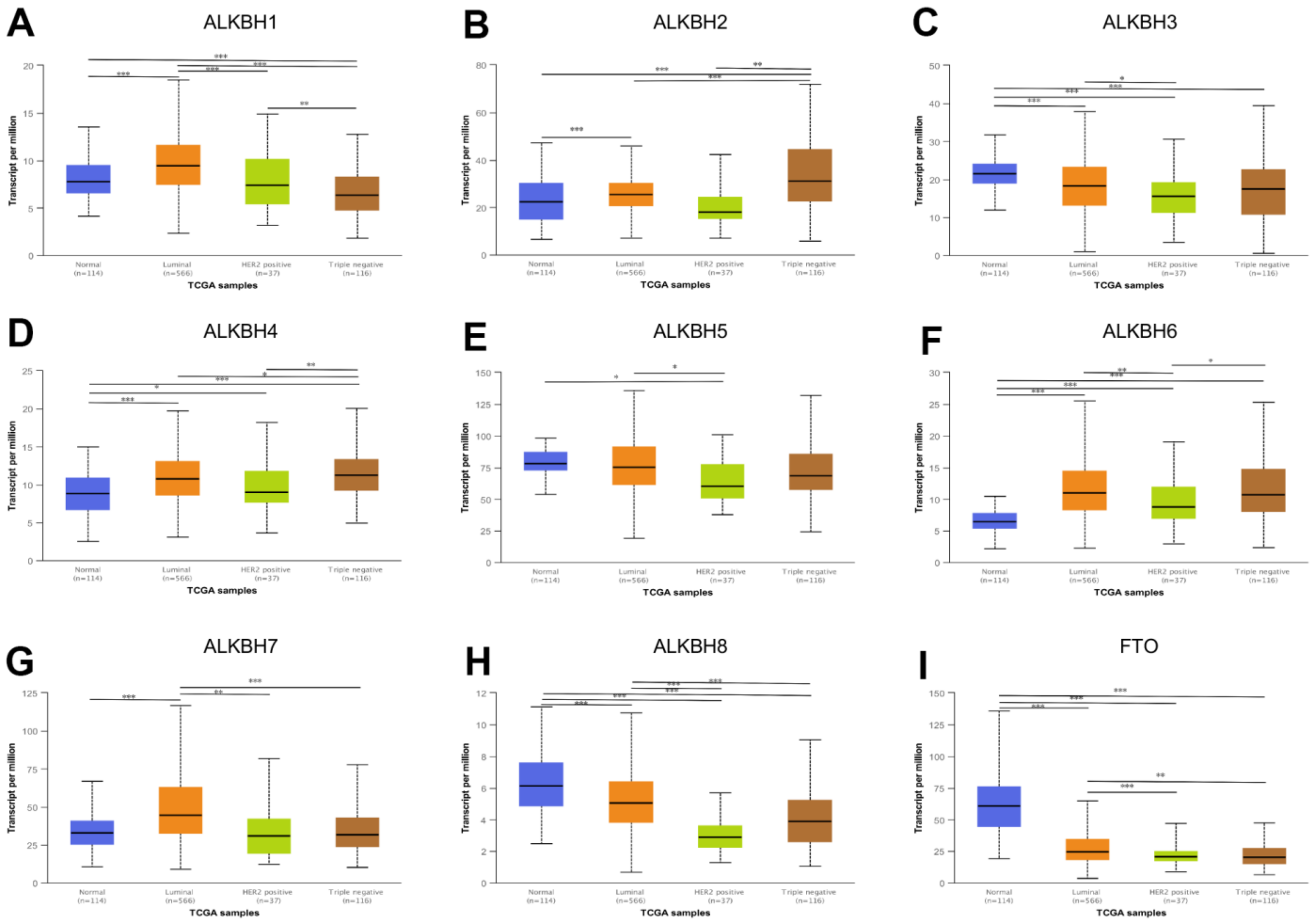

Supplementary Figure 1. The correlation between the ALKBH family and major subclasses of breast cancer. (**A**–**I**) The relationship between ALKBH1-8 and FTO mRNA expression levels and major subclasses in BRCA (UALCAN). *: P<0.05, **: P<0.01, ***: P<0.001.
